# Human myoma tissue-based extracellular matrix models for testing the effects of irradiation on the HPV positive cells

**DOI:** 10.1186/s12985-020-01367-1

**Published:** 2020-06-30

**Authors:** Heidi Tuominen, Ahmed Al-Samadi, Tuula Salo, Jaana Rautava

**Affiliations:** 1 Department of Oral Pathology and Oral Radiology, Institute of Dentistry, Faculty of Medicine, University of Turku, Lemminkäisenkatu 2, FIN-20520 Turku, Finland; 2 Department of Pathology, Turku University Hospital, Turku, Finland; 3 Translational Immunology Program, Faculty of Medicine, University of Helsinki, Helsinki, Finland; 4 Department of Oral and Maxillofacial Diseases, University of Helsinki, Helsinki, Finland; 5 Medical Research Center Oulu, Oulu University Hospital, Oulu, Finland; 6 Cancer Research and Translational Medicine Research Unit, Faculty of Medicine, University of Oulu, Oulu, Finland; 7 HUSLAB, Department of Pathology, Helsinki University Central Hospital, University of Helsinki, Helsinki, Finland

**Keywords:** HPV, Cell culture, Cell invasion, Ionizing radiation, Myogel, Myoma

## Abstract

**Background:**

This study was designed to investigate the invasion of human papillomavirus (HPV) positive human cervical carcinoma cell lines in human leiomyoma-based extracellular matrices in vitro*,* and to test the suitability of the model for studying the irradiation effects on the cancer cell invasion.

**Methods:**

HPV positive cervical carcinoma cell lines SiHa and CaSki, and HPV negative squamous cell carcinoma cell line HSC-3 were used. CaSki cells contain around 600 copies of HPV 16 virus in the genome, whereas SiHa have only 1–2 copies per cell. Cells were analyzed using two different human tumor derived extracellular matrix methods (3D myoma disc model, and Myogel Transwell invasion assay). Cultures were irradiated with 4 Gy. Myoma invasion area and the depth of invasion were measured with ImageJ 1.51j8 software. Statistical analyses were performed with SPSS Statistics (IBM SPSS® Statistics 25).

**Results:**

All cells invaded through Myogel coated Transwell membranes and within myoma discs. In myoma discs, a difference in the invasion depth (*p* = 0.0001) but not in invasion area (*p* = 0.310) between the HPV positive cell lines was seen, since SiHa (less HPV) invaded slightly better than CaSki (more HPV). HSC-3 cells (HPV negative) invaded deepest (*p* = 0.048) than either of the HPV positive cell line cells. No difference was detected in the invasion area (*p* = 0.892) between HPV positive and HPV negative cells. The ionized radiation significantly reduced the invasion depth of HSC-3 (*p* = 0.008), SiHa (*p* = 0.0001) and CaSki (*p* = 0.005). No significant effect on the invasion area was detected in any of the cell lines. However, a significant difference was observed between SiHa and CaSki in the reduction of the invasion depth after radiation (*p* = 0.013) as the reduction was greater with SiHa than CaSki.

**Conclusions:**

Both solid and gelatinous human leiomyoma-based extracellular matrix models were suitable platforms to study the invasion of HPV positive cervical carcinoma cells in vitro. SiHa cells with less HPV copy number cells invaded slightly better and were slightly more sensitive to irradiation than CaSki cells with high HPV copy number. However, there was no drastic differences between the invasion properties of these carcinoma cells.

## Background

In vitro models are important tools for cancer research. For cell cultures, the most commonly used matrix over the past decades has been Matrigel® (BD Matrigel Matrix, BD Biosciences, New Jersey, United States) which is derived from mouse sarcoma [1]. However, in order to better understand human cancer cells interaction with human tumor matrices there has been a need for human tumor tissue-derived matrices. Recently, a 3D solid in vitro human uterus leiomyoma tissue derived disc model has been developed for invasion analyses followed by a gelatinous Myogel matrix for attachment, Transwell and spheroid invasion assays [1–6]. Myoma discs offer all the main parts of tumor microenvironment; such as vessels, collagen fibers and soluble factors, including cytokines and growth factors [4, 7–10]. This provides us with better knowledge of cancer cells behavior in vivo. HSC-3 is an aggressively invading HPV negative squamous cell carcinoma cell line which has been extensively studied in both myoma discs and Myogel assays [2, 8, 11–15].

Persistent infection with high-risk (HR) human papillomavirus (HPV), mainly with HPV 16, is a major cause of cervical squamous cell carcinomas (CSCC) and increasingly also oropharyngeal carcinoma [16]. SiHa and CaSki are two HPV 16 positive commercially available carcinoma cell lines derived from human squamous cell carcinomas of cervical uteri. CaSki cells contain approximately 600 viral copies per cell, whereas SiHa has less than ten copies per cell [17, 18]. CaSki also contains some sequences related to HPV 18 [19]. Cervical carcinoma cell lines have not previously been investigated in human tumor-based extracellular matrices in vitro.

Irradiation treatment may be beneficial or harmful for the cancer patient. HPV positive carcinomas are shown to be more radiosensitive than HPV negative carcinomas and the patients have a better prognosis and treatment outcome [20–26]. The HPV negative CSCCs have also been shown to have greater potential to metastasize (with aggressive p53 mutations), which would explain the worse prognosis and poorer growth control without radiation treatment [26–28]. Based on this controversy, we wanted to investigate if the in vitro human myoma tissue-based extracellular matrix models are suitable for testing the radiation impact on the HPV positive carcinoma cells. HPV negative cells was used as a positive control in this study.

## Methods

### Cell lines

Two HPV positive squamous cell carcinoma lines SiHa (ATCC® HTB-35™) (LGC Standards GmbH, Wesel, Germany) and CaSki (ATCC® CRL-1550™) (LGC Standards GmbH, Wesel, Germany) were used for cell cultures. SiHa and CaSki are HPV 16 positive carcinoma cell lines. CaSki cells contain approximately 600 viral copies of HPV 16 per cell and SiHa less than ten copies per cell [17, 18]. As a positive control, a HPV negative human oral squamous cell carcinoma cell line HSC-3 (JCRB 0623; Osaka National Institute of Health Sciences, Osaka, Japan) with known aggressive behavior was used [2, 8, 11–15].

The SiHa and CaSki were cultivated in DMEM with 10% heat-inactivated fetal bovine serum (FBS), 100 U/ml penicillin, 100 μl/ml streptomycin and 1% non-essential amino acids (all from Gibco, Life Technologies, Paisley, UK). HSC-3 cells had a different media, which consisted of DMEM/F-12 (Dulbecco’s modified Eagle’s media/F12, 1:1) with 10% heat-inactivated fetal bovine serum (FBS), 100 U/ml penicillin, 100 μl/ml streptomycin (Gibco, Life Technologies, Paisley, UK) in addition with 4 mg/ml Hydrocortisone, 50 μg/ml Ascorbic Acid (vitamin C) and 250 ng/ml Fungizone (Sigma-Aldrich, Ayrshire, UK).

### Human extracellular matrix models

The human uterine leiomyoma–based solid matrix model, the myoma disc model, has been previously described in detail in Nurmenniemi et al. [4]. Briefly, the myomas have been obtained from routine surgical procedures after obtaining the patients’ informed consent. The original study was approved by the Ethics Committee of the Oulu University Hospital (statement #8/2006, amendment 19/10/2006 and statement #35/2014, 28.4.2014). The myoma discs were cut from 3 mm slices with 8 mm biopsy punch and placed in − 70 °C in cell culture media with 10% DMSO (Sigma-Aldrich) for storage prior to be used in invasion experiments.

The gelatinous leiomyoma matrix, Myogel, has been prepared as described earlier [3]. Briefly, the protocol is similar to the Matrigel® preparation with slight modifications [29]. The liquid nitrogen frozen myoma tissue from several patients were combined, grounded to powder, suspended to NaCl buffer, homogenized, and the protein concentration was matched to that of Matrigel®. Myogel preparations were stored in − 20 °C.

### Cell viability testing

Cell viability tests were performed to all cell lines cultured either on top of the Myogel or on top of plastic wells. For Myogel assay, the 96-well Transwell plate was coated with 50 μl Myogel (0.5 mg/ml) and left to settle overnight in the cell culture incubator. The excess of the coating was removed carefully with suction, and 1000 cells in 100 μl media per well were added. The cells were allowed to grow for 72 h at + 37 °C. CellTiter-Glo® (CTG) 2.0 Luminescent Cell Viability Assay (Promega, Madison, WI, USA) was used to determine the cells viability. The plate was taken out from the incubator to room temperature for 30 min before starting the assay. One hundred microliters of CellTiter-Glo was dispensed in each well. The plate was put on a plate shaker (Heidolph, Schwabach, Germany) for 5 min at 450 rpm and then in plate spinner (Thermo Scientific, Massachusetts, USA) for 5 min at 1000 rpm. The plate was then placed in the BMG PHERAstar FS (BMG Labtech, Offenburg, Germany) plate reader to detect cell viability. The results represent an average of three independent experiments.

### Myogel Transwell invasion assay

For the Myogel Transwell invasion assay, a mix of Myogel with 0.8 mg/ml rat collagen and 2.4 mg/ml Myogel was prepared. A 50 μl of this Myogel mix was used to coat 24-well Transwell plate, followed by 30 min to 1 h incubation at + 37 °C for the gel to develop. A volume of 500 μl of normal cell culture media was added to the bottom of the well. Total of 70,000 cells in 200 μl lactalbumin media were added on top of Myogel. In lactalbumin media, the FBS was replaced by 0.5% lactalbumin (Sigma-Aldrich).

The cells were allowed to grow and invade for 72 h and then fixated with 4% formaldehyde for 1 h and washed with PBS. Invaded cells were stained with 1% Toluidine blue + 1% Borax at room temperature. After staining, the cells were washed several times with distilled water and non-invaded cell from the upper chamber were carefully removed with wet cotton swabs. Staining was eluted with 1% Sodium Dodecyl Sulfate (SDS) and transferred to 96-well plate. The absorbance was measured at 650 nm with Synergy™ HT (BioTek Instruments, Inc., Winooski, Vermont, USA) according to manufacturer’s instructions. The results represent an average of three cultures, performed in triplicate.

### Myoma invasion assay and irradiation

To prepare the cultures, the myomas were thawed to room temperature and washed with appropriate media. Myoma discs were placed in tubes with 10 ml media to wash overnight. After 24 h, myomas were placed in a 24-well Transwell inserts (diameter, 6.5 mm; Corning, Inc., Corning, NY). Around 750,000 SiHa or CaSki or HSC-3 cells in 50 μl media were added on top of each myoma disc. Lower chamber of the well was filled with 0.5 ml cell culture media. Cells were allowed to attach and grow for 24 h in + 37 °C. After this, the myoma discs were removed from the inserts and placed in 12-well Transwell on top of metal grid and a nylon membrane with 1 ml media in the bottom of the well. The cells were allowed to invade for 13 days. The cell culture media was changed in every 3 to 4 days. The cultures were finished on day 14, and the tissues were prepared for histology.

Faxitron MultiRad350 (DatekMedíco, Aarhus, Denmark) irradiator was used according to manufacturer’s instructions for a part of the myomas (one 12-well plate) in order to give the cells ionized radiation equivalent to 4 Gy on the third day of cultivation. One 12-well plate Transwell with the cells was similarly carried around and mock-irradiated simultaneously.

Hematoxylin-eosin (HE) staining was used to estimate the overall success of the cell culture experiment, and cytokeratin staining (Cytokeratin High Molecular Weight, Clone 34BE12, Dako, Clostrup, Denmark) was used to analyze the invasion depth and invasion area of the cells (see below). All experiments were performed in triplicates, representing an average of two to six independent cultures.

### Cell invasion analyses

Fiji ImageJ 1.52a (National Institute of Health, USA) software was used to analyze and calculate the invasion depth and the invasion area of the myoma invasion model cultured with SiHa, CaSki or HSC-3 cells, as described in detail in Åström and co-workers [5]. The invasion index (%) depicts a proportion of an area where cells have invaded in percentage and is calculated as epithelium area divided by the total area of invaded cells area. The invasion area describes the overall area (μm^2^) of invaded cells. The average of invasion depth (μm) depicts how deep the cells have been able to invade into the tissue.

The statistical analyses of all the results were performed with SPSS Statistics (IBM SPSS Statistics version 21). After the normality testing of the data, for unrelated samples, either the independent samples Student’s T-test or Mann-Whitney’s U-test were used to determine statistical differences. If the samples were related, either paired samples t-test or Wilcoxon signed-rank test were used. In all calculations, a value of *p* < 0.05 was considered statistically significant.

## Results

### Cell viability assay

**Fig. 1:**
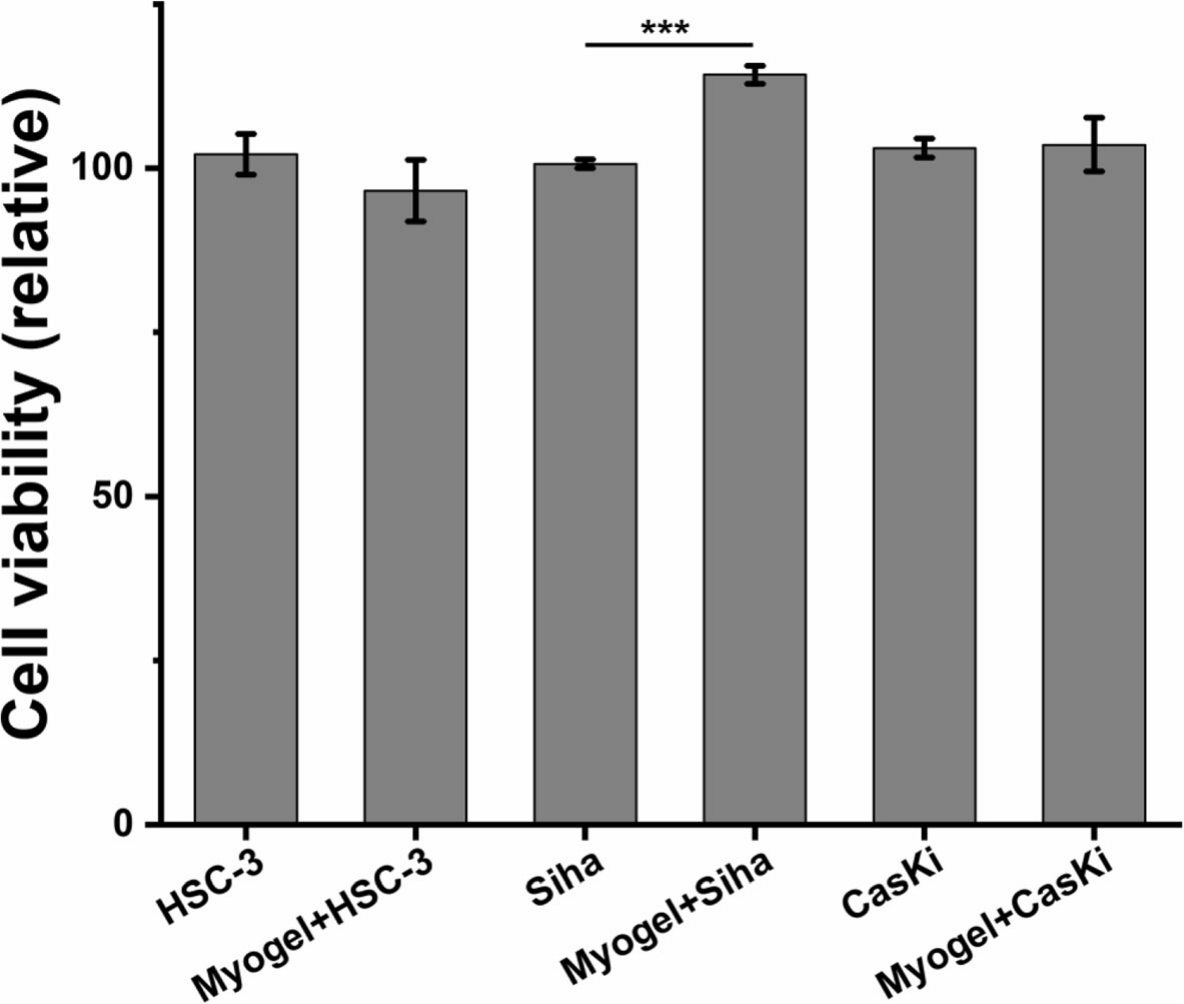
Cell viability. SiHa cells on Myogel presented better viability when compared to the control. Other cell lines did not have differences between Myogel and control. ***p < 0.001

All three cell lines survived in Myogel extract coated wells (Fig. 1). SiHa cells showed significantly higher viability when cultured on Myogel coated wells compared with plastic (*p* = 0.0001). No differences were observed in the viability of either CaSki or HSC-3 when compared controls to Myogel.

### Myogel Transwell invasion assay

**Fig. 2:**
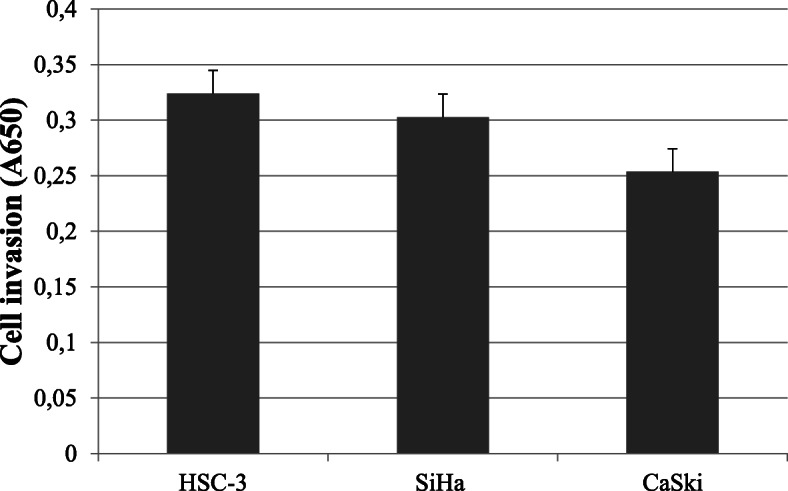
Myogel Transwell invasion assay. The absorbance values for Myogel Transwell invasion were 0.32 for HSC-3, 0.31 for SiHa and 0.25 for CaSki. No statistically significant differences (*p* < 0.05) were observed in Myogel Transwell invasion between the different cell lines

In the Myogel Transwell invasion assay (Fig. 2), all the cell lines invaded through the Myogel mixture. We detected no statistically significant differences in the results according to the different cell lines (HSC-3 and SiHa *p* = 0.406, HSC-3 and CaSki *p* = 0.406, SiHa and CaSki *p* = 0.963).

### Myoma invasion assay

**Fig. 3:**
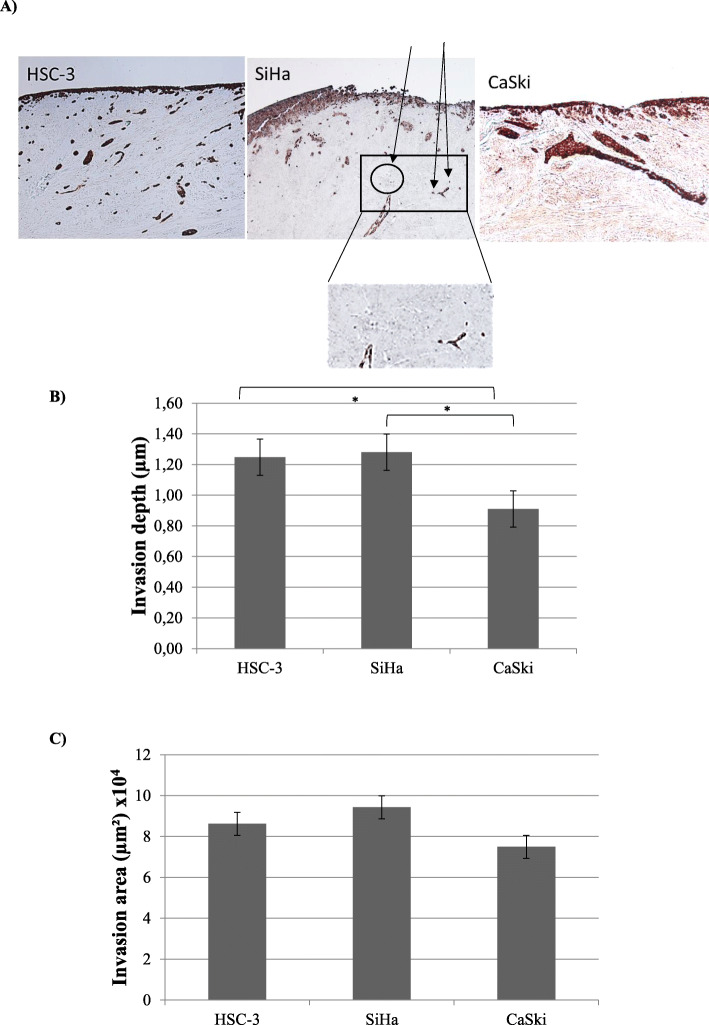
Myoma invasion assay. The Cytokeratin stained figures of HSC-3, SiHa and CaSki (**a**) are presented at 50 x magnification. Arrows have been added in SiHa to represent the small invaded islet (“buds”), also the area shown has been enlarged below to clearly depict the invasion. The invasion depth (**b**), was 1.25 μm in HSC-3, 1.28 μm in SiHa and 0.91 μm in CaSki. The difference between SiHa and CaSki was statistically significant (*p* < 0.01) in the invasion depth without irradiation. The invasion area (**c**) had also some differences. The invasion area in HSC-3 was 86.2 mm^2^, 94.3 mm^2^ in SiHa and 74.9 mm^2^ in CaSki. Significant differences were observed between HSC-3 and CaSki (*p* < 0.05) and SiHa and CaSki (*p* < 0.05). The statistically significant differences have been marked in the figures by adding the asterisks (*p ≤ 0.001)

All three cell lines invaded within myoma discs (Fig. 3a). In the invasion depth, SiHa and CaSki had a statistically significant difference (*p* = 0.0001). The difference in the invasion depth was also significant between HSC-3 and CaSki (*p* = 0.003), but not between SiHa and HSC-3 (*p* = 0.602). HPV negative HSC-3 invaded slightly better than HPV positive (SiHa and CaSki) cells combined (*p* = 0.048). The invasion depth of all three cell lines are presented in Fig. 3b.

Considering invasion area (Fig. 3c), no significant differences were detected between the cell lines (HSC-3 and SiHa *p* = 0.937, HSC-3 and CaSki *p* = 0.699 and SiHa and CaSki *p* = 0.310). No differences were neither observed in invasion area based on the HPV status (*p* = 0.892).

The invasion index with HSC-3 reached up to 53.6%. SiHa and CaSki presented more modest invasion index of 21.3 and 30.0%. These differences did not reach statistical significance.

### Irradiation effect to cell invasion

**Fig. 4:**
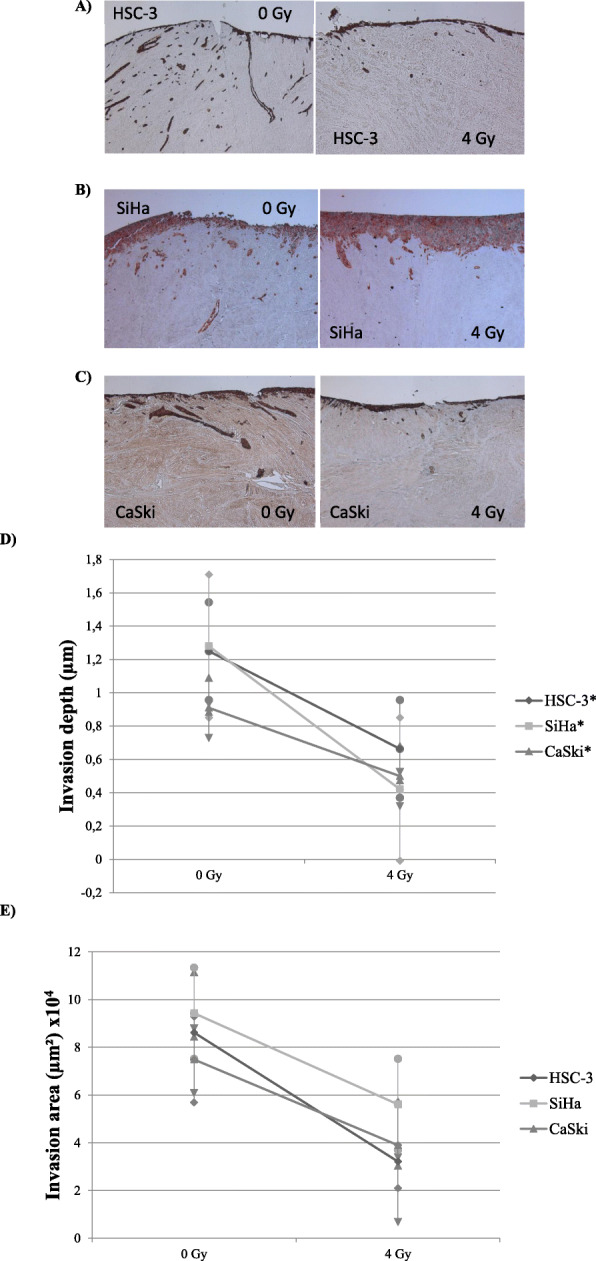
Irradiation effect to cell invasion. Cytokeratin stained sections at × 50 magnification before (0 Gy) and after (4 Gy) irradiation in the HSC-3 (**a**), SiHa (**b**) and CaSki (**c**) are presented. After irradiation the invasion depth decreased from 1.25 μm to 0.66 μm in HSC-3 (*p* < 0.001), from 1.28 μm to 0.42 μm in SiHa (*p* < 0.001) and from 0.91 μm to 0.50 μm in CaSki (*p* < 0.01). Also, the invasion area was decreases in HSC-3 from 86.2 mm^2^ to 32.1 mm^2^, SiHa from 94.3 mm^2^ to 56.1 mm^2^ and CaSki from 74.9 mm^2^ to 39.0 mm^2^, but not with statistical significance. The differences in invasion depth (**f**) and invasion area (**g**) were measured and compared before (0 Gy) and after (4 Gy) irradiation. Statistically significant differences have been marked to figures as *p ≤ 0.001

After a single 4 Gy irradiation, the invasion depth was significantly reduced in irradiated HSC-3 (from 1.25 μm to 0.66 μm, *p* = 0.008, Fig. 4a), SiHa (from 1.28 μm to 0.42 μm, *p* = 0.0001, Fig. 4b) and CaSki (from 0.91 μm to 0.50 μm, *p* = 0.005, Fig. 4c) cell myoma tissue sections when compared to the mock-radiated myoma sections (Fig. 4d). Between the two HPV positive cell lines, the invasion decreased significantly more in SiHa than in CaSki cells (*p* = 0.013).

No statistically significant difference was observed in the reduction of invasion area after radiation in HSC-3 (from 86.2 mm^2^ to 32.1 mm^2^, *p* = 0.109), in SiHa (from 94.3 mm^2^ to 5.61 mm^2^, *p* = 0.109) or in CaSki (from 74.9 mm^2^ to 3.90 mm^2^, *p* = 0.109) despite the major differences in the invasion depth after irradiation (Fig. 4e). No differences were neither observed between the HPV positive cervical carcinoma cell lines in the reduction of invasion area (*p* = 0.222).

The calculated invasion indexes decreased significantly in all cell lines (HSC-3 53.6% without irradiation and 29.5% after (*p* = 0.003); SiHa 21.3% without irradiation and 13.7% after (*p* = 0.003) and CaSki 30.0% without irradiation and 26.5% after (*p* = 0.006)) as demonstrated in the statistically significant differences in the invasion depths as well.

## Discussion

Human extracellular matrices, myoma discs and Myogel, presented as useful study platforms for cervical carcinoma cell lines invasion, since both SiHa and CaSki cells invaded these matrices and 4 Gy irradiation significantly reduced their invasion in myoma disc experiment.

We have introduced gelatinous Myogel Transwell invasion assay, which has proved to be a fast method for cancer cells invasion experiments [1, 3, 6, 7, 30]. Carcinoma cells invade faster in Myogel than in the traditionally used commercial Matrigel® [4, 6]. Viability was good in all of the cell lines. Nevertheless, SiHa with Myogel performed significantly better than without Myogel (*p* = 0.0001). Similar differences were not observed with CaSki or HSC-3. Furthermore, in our Myogel Transwell invasion assay there were no differences between cell lines in the invasion through the Myogel mixture; invasion was detected similarly in both of the HPV positive cervical carcinoma cell lines.

The myoma disc model closely mimics the human tumor microenvironment of solid cancers [1, 2, 8, 9]. Here we detected major differences between SiHa (less HPV) and CaSki (more HPV) in the invasion depths, as SiHa invaded deeper and more aggressively. They are both cervical squamous cell carcinoma cell lines, but each neoplasia is individual. We cannot rule out the possibility of differences between the cell lines themselves. However, this may also reflect to the difference between the viral copy numbers with the carcinoma cells. Nonetheless, the difference was not observed in the invasion area. This may, at least partly, be explained by the different invasion patterns on cellular level. SiHa cells invaded either as single cells or with small islands (buds) deeper into the myoma tissue while CaSki formed bigger invasive cell droplets and invaded less deep than SiHa cells (Fig. 3a).

The carcinoma cell invasion budding, as detected in SiHa invasion to myoma, have independently been connected in clinical studies with deeper invasion depth, venous invasion, lymph node metastasis and poor prognosis in head-and-neck squamous cell carcinoma (HNSCC) and colorectal cancer [31–40]. Also, both in cervical adenocarcinomas and CSCCs, tumor budding has been associated with higher histological tumor grade, invasive pattern, lymph node metastasis, bigger tumor size and poorer prognosis [41, 42]. Jesinghaus and collagues [42] have suggested in their study, that tumor budding could be used as an individual prognostic factor in CSCCs. There have not been similar studies concerning HPV positive carcinomas in either HNSCCs or CSCCs, but we would speculate that similar effect might be seen regardless of the HPV status, as was observed with HPV positive SiHa cell in our study.

Since significant differences were observed in myoma discs between different cell lines, we further wanted to investigate the impact of ionized radiation to cell invasion. We have shown in the previous myoma experiments that 4 Gy radiation is optimal dose to for the major changes in various oral carcinoma cell lines invasion [30]. Here we detected that the invasion depth was significantly reduced in all experiments with dose of 4 Gy, which was in line with our previous experiment [30]. We also showed that ionized radiation affected to the total invasion area, and the invasion index decreased clearly in all of the cells studied: 27.1% in HSC-3, 7.6% in SiHa, and 3.5% in CaSki cells. These results suggest, that ionized radiation reduces, not only the invaded cells invasion depth into the tissue, but also the overall volume of the invaded cell islets, decreasing the aggressive potential of all the carcinoma cells. These findings suggest that even a relatively small radiation dose impacts to the cell invasion.

Between HPV positive cervical cell lines, a difference was observed regarding the reduction of invasion after irradiation, as SiHa with less HPV, the reduction was greater than with CaSki. HPV positive CSCC and HNSCC are known to have more favorable prognosis and treatment outcome than HPV negative [23, 26, 43]. HPV negative and HPV positive CSCC patients with multiple simultaneous infections have been observed to have poorer prognosis even after radiation treatment [24, 25]. No difference to radiation between HPV positive and HPV negative cell lines in HNSCCs have been observed [44, 45], but still one study have presented higher radiosensitivity of HPV positive HNSCC cells [46]. Based on our study, HPV copy number had only a modest effect on the radiosensitivity of the cell invasion. However, the trend was that the radiation was more effective towards non (HSC-3) and less HPV infected (SiHa) carcinoma cells than in case of high copy number of HR-HPV 16 (CaSki).

Our study has some limitations. Although the myoma discs provides an excellent hypoxic, human tumor derived 3D solid platform to study cancer cells behavior in vitro, it is essential to keep in mind that human body has several other factors, such as blood derived cells and cytokines that affect cancer cells aggressiveness in vivo*.* As a technique, the use of Myogel Transwell invasion assay is quite sensitive and multiple repetitions are needed in order to produce secured results. Furthermore, we only used three different cell lines, two HPV positive and only one HPV negative, and here we present novel results on the HPV positive cells behavior in myoma and Myogel assays. The differences could be explained by individual nature of each cell line. SiHa and CaSki cell lines are HPV 16 positive cervical cancer cell lines but have different origins and CaSki even might contain some sequences of HPV 18 [17–19]. The results presented here shows how these cell lines interact with myoma and Myogel and that the cell invasion is evident in all three different cases. We have used oral squamous cell carcinoma cell line HSC-3 as a control in our experiments, since it has most widely been used in Myoma and Myogel studies previously [2, 8, 11–15]. Therefore, we already have a good knowledge of its behavior in these cell matrices and it offers us the best way to compare between the different cell lines. Furthermore, similar outcomes were observed between different cell lines in different experiments.

## Conclusions

Solid and gelatinous human uterine leiomyoma-based extracellular matrix models were usable platforms to study the invasion of HPV positive cervical carcinoma cells in vitro. Carcinoma cells with less HPV copy number (SiHa) invaded slightly better and were slightly more sensitive to irradiation than cells with a high copy number (CaSki).

## Data Availability

The dataset used and/or analyzed during the current study are available from the corresponding author on reasonable request.

## Abbreviations

CSCC: Cervical squamous cell carcinoma
DMEM: Dulbecco’s modified Eagle’s medium (minimal essential medium)
DMSO: Dimethyl sulfoxide
FBS: Foetal bovine serum
HNSCC: Head-and-neck squamous cell carcinoma
HPV: Human papillomavirus
HR-HPV: High-risk human papillomavirus
SDS: Sodium Dodecyl Sulphate
